# A Multi-Specific DARPin Potently Neutralizes Shiga Toxin 2 via Simultaneous Modulation of Both Toxin Subunits

**DOI:** 10.3390/bioengineering9100511

**Published:** 2022-09-27

**Authors:** Yu Zeng, Mengqiu Jiang, Sally Robinson, Zeyu Peng, Vikas Chonira, Rudo Simeon, Saul Tzipori, Junjie Zhang, Zhilei Chen

**Affiliations:** 1 Department of Microbial Pathogenesis and Immunology, Texas A&M University Health Science Center, 8847 Riverside Pkwy, Bryan, TX 77807, USA; 2 Center for Phage Technology, Department of Biochemistry and Biophysics, Texas A&M University, 300 Olsen Blvd., College Station, TX 77843, USA; 3 Department of Infectious Disease and Global Health, Cummings School of Veterinary Medicine, Tufts University, 200 Westboro Rd, North Grafton, MA 01536, USA; 4 Interdisciplinary Graduate Program in Genetics, Texas A&M University, 300 Olsen Blvd., College Station, TX 77843, USA

**Keywords:** dysentery, therapy, antibiotic, biologic, kidney

## Abstract

Shiga toxin-producing *E. coli* (STEC) is a common cause of bloody diarrhea. The pathology of STEC infection derives from two exotoxins—Shiga toxin 1 (Stx1) and Shiga toxin 2 (Stx2)—that are secreted by STEC in the gut, from where they are systemically absorbed, causing severe kidney damage leading to hemolytic uremic syndrome (HUS). Currently, there is no effective treatment for HUS, and only supportive care is recommended. We report the engineering of a panel of designed ankyrin repeat proteins (DARPin) with potent neutralization activity against Stx2a, the major subtype associated with HUS. The best dimeric DARPin, SD5, created via a combination of directed evolution and rational design, neutralizes Stx2a with a half maximal effective concentration (EC_50_) of 0.61 nM *in vitro.* The two monomeric DARPin constituents of SD5 exhibit complementary functions—SHT targets the enzymatic A subunit of Stx2a and inhibits the toxin’s catalytic activity, while DARPin #3 binds the B subunit, based on the cryo-EM study, and induces a novel conformational change in the B subunit that distorts its five-fold symmetry and presumably interferes with toxin attachment to target cells. SD5 was fused to an albumin-binding DARPin, and the resulting trimeric DARPin DA1-SD5 efficiently protects mice in a toxin challenge model, pointing to a high potential of this DARPin as a therapeutic for STEC infection. Finally, the unprecedented toxin conformational change induced by DARPin #3 represents a novel mode of action for neutralizing Stx2 toxicity and reveals new targets for future drug development.

## 1. Introduction

Shiga toxin (Stx)-producing *Escherichia coli* (STEC) is one of the most common infectious causes of bloody diarrhea, also known as hemorrhagic colitis (HC), with an estimated 265,000 STEC infections in the United States each year [1]. Infection occurs when patients ingest contaminated food (e.g., salad and under cooked meats) or water (e.g., drinking water or swimming pool water), or by direct contact with infected animals or humans [2]. Although GI dysfunction is the most visible symptom of the infection, a serious complication of STEC infection is hemolytic uremic syndrome (HUS), which is characterized by acute renal failure, thrombocytopenia, and microangiopathic hemolytic anemia, which occurs in 4−15% of STEC infection cases, mostly those affecting children and the elderly [3,4,5]. About 40% of the patients with HUS develop chronic complications [6]. The largest documented outbreak of STEC infection worldwide occurred in Germany in 2011, affecting 3816 patients and causing 54 deaths. Among these, 845 patients developed HUS [7].

Currently, there is no effective treatment for STEC infection. The pathology of STEC infection is primarily caused by the action of two exotoxins—Shiga toxin 1 (Stx1) and Shiga toxin 2 (Stx2)—which attack the ribosomes and trigger apoptosis upon their uptake by the host cells [8]. Intimin, an adhesin molecule, is another important virulence factor that facilitates bacterial attachment to colonocytes. Although antibiotic treatment can reduce the STEC load, it also increases Stx-phage synthesis and Shiga toxin release, leading to an increased risk of developing HUS and death [9,10,11,12]. Consequently, CDC guidelines recommend that persons infected with STEC be provided with only supportive care—oral and IV fluids and pain control [13]—and not be treated with antibiotics [14]. Five predominant Stx1 subtypes and ten Stx2 subtypes have been reported [15,16], with Stx2a, Stx1a, and Stx2c mainly associated with HUS [17,18,19,20,21]. Stx2 exhibits higher toxicity than Stx1 in both mice and humans [22].

Shiga toxins belong to the AB_5_ family of protein toxins and are comprised of an enzymatically active A subunit and a non-toxic pentameric B subunit [23,24]. Interaction between the B subunit and the carbohydrate moiety of the glycosphingolipid Gb_3_ on the extracellular leaflet of cell plasma membranes enables these toxins to enter host cells through receptor-mediated endocytosis and to be retrogradely transported from the Golgi apparatus to the endoplasmic reticulum (ER) [25,26,27,28]. Some Shiga toxins are able to cross the epithelial cell barrier and enter the circulatory system, from where they travel to the kidneys and damage Gb_3_-expressing glomerular endothelial cells, causing the onset of HUS [3].

In recent years, a number of Shiga toxin-neutralizing antibodies have been developed as therapeutics for STEC [29,30,31,32,33,34]. Unfortunately, clinical evaluation of these anti-toxin antibodies has been slow, largely due to the logistics and cost involved. Antibody production requires mammalian cell culture, which is expensive to maintain. The sporadic nature of STEC infection, combined with the limited shelf life and high cost of antibodies, dampens the enthusiasm of many hospitals to stockpile anti-toxin antibodies and thwarts the willingness of drug companies to develop antibody-based STEC therapeutics.

To circumvent the high cost associated with antibody therapeutics, we engineered alternative toxin-neutralizers templated on the designed ankyrin repeat protein (DARPin). DARPin is a small (18 kDa) scaffold protein which has been engineered to bind diverse targets [35], and it enjoys a low immunogenicity potential, a very high expression yield in microorganisms (15 g per liter of *E. coli* culture in fermenters, or 23% of dried cell weight) [35,36,37], high solubility, and long shelf life [36]. The high thermostability and high microbial expression yield make DARPin an attractive alternative to antibody therapeutics.

Employing a protein engineering approach combining both directed evolution and rational design, we created a dimeric DARPin SD5 with potent neutralization activity against Stx2a (EC_50_ 0.6 nM). SD5 is composed of two DARPins—SHT, which targets the toxin A subunit and neutralizes the toxin catalytic activity, and #3, which binds the B subunit and induces a novel conformational change in the B subunit that distorts the five-fold symmetry and interferes with toxin attachment. DA1-SD5, composed of SD5 fused to an albumin-binding DARPin [38], efficiently protects mice against Stx2a intoxication when premixed with the toxin. These studies support the further preclinical and clinical evaluation of DARPin DA1-SD5 for preventing the HUS complication of STEC infection.

## 2. Materials and Methods

### 2.1. Protein Expression and Purification

Stx2a, Stx2a-A1, and Stx2a-B were recombinantly expressed in *E.coli* BL21(DE3) cells and purified via the use of a nickel-nitrilotriacetic acid (Ni-NTA) column. The holotoxin Stx2a was concentrated and buffer exchanged into phosphate-buffered saline (1xPBS, pH 7.4) using the Amicon ultrafiltration unit (MWCO 50 kDa). Protein purity was confirmed using SDS-PAGE. The concentration of purified protein was determined by BCA assay (Thermo Scientific Pierce BCA Protein Assay, Fisher catalog no. PI23227). The purified protein was stored at −20 °C in 50% glycerol. (Figure S1). Purified Stx2c was obtained from BEI Resources (Cat# NR-13422).

### 2.2. DARPin Library Construction

An in-house prepared DARPin library with ~2 × 10^9^ variants was used in the initial phage panning [39]. For the saturation mutagenesis, the selected residues were replaced with the NNK codon. The resulting library was cloned into the pET28a vector and transformed into Bl21(DE3) cells for protein expression and functional screening. For the off-rate selection library, error-prone PCR was used to introduce random mutations to selected monomer DARPins (i.e., 10B, HT, RI, #1, #3, #9, #12, #16, and #20). The mutation rate was determined to be 3.7 nucleotide changes per gene based on the sequencing result. This library was displayed on the M13 phage as an N-terminal fusion to gIII and used in phage panning. The dimeric DARPin library was created essentially as described previously [39,40], except that a (G_4_S)x4 linker was used to connect the two DARPins.

### 2.3. Phage Panning

Phage panning was carried out essentially as described previously [39,40]. Holotoxin Stx2a was biotinylated using EZ-Link-Sulfo NHS-LC biotin (Pierce) and used as the target in phage panning. DARPin variants from the 3rd round of phage panning were cloned into the pET28a vector (containing an N-terminal His tag and a Myc tag). A total 752 different colonies of *E. coli* Bl21(DE3) cells transformed with the enriched DARPin library were selected and grown in 96-well plates.

For off-rate selection, a previously published protocol was used, with minor modifications [41,42]. Briefly, the phage library (~10^12^ phage particles) was first incubated with biotinylated Stx2a (1 nM) in 1 mL of PBS overnight at 4 °C. The next day, a 500-fold molar excess of non-biotinylated Stx2a (500 nM) was added, and this mixture was incubated with slow shaking for 4, 16, and 140 h at room temperature for rounds 1, 2, and 3, respectively. Phage particles that remain bound to the biotinylated toxin were enriched for variants with a slower off-rate and were recovered using Dynabeads MyOne streptavidin-coated magnetic beads (Thermofisher Scientific, Waltham, MA, USA). To ensure the enrichment of DARPin molecules able to remain associated with Stx2a upon translocation into the acidic endosome, the beads were thoroughly washed in both regular and acidic PBS+ 0.1% Tween-20 (PBST) buffer (pH 4.5, buffered with 10 mM sodium citrate) before the elution of the bound phage.

### 2.4. Functional Screening

The expressed DARPins were semi-purified via lysozyme and heat treatment [40], and the toxin neutralization potency of each selected DARPin was evaluated using the Vero or Vero-E6 cell toxin challenging assay. For the initial monomer DARPin screening, Vero cells were seeded the night before in 96-well plate (1.5 × 10^3^ cells/well, 100 μL/well) in complete growth medium supplemented with 10% FBS and 1× anti-antibiotic antimycotic (Life Technologies catalog no. 15240062). The next day, the soluble cell lysate (20 μL/well) and Stx2a (80 μL, final 10 pg/mL) were added to the Vero cells (total 200 μL/well). The plates were incubated at 37 °C and 5% CO_2_ for 72 h, and the cell viability was quantified using the CellTiter-Glo reagent (Promega) and normalized to that of the naïve Vero cells.

For dimer library and the off-rate library screening, Vero E6 cells were used instead. A mixture of Vero E6 cells (100 μL, 1.5 × 10^3^ cells/well), the soluble lysate (20 μL/well), and diluted Stx2a (80 μL, final 10 pg/mL or 0.1 pM) were seeded simultaneously in 96-well plates. The cell viability was quantified, as described above.

### 2.5. Protein Purification

Candidate DARPin clones were purified by standard metal chelation chromatography, as previously described [40]. For the in vivo experiment, the eluted protein was first buffer-exchanged into DPBS (Fisher, Waltham, MA, USA, cat SH30028FS) and passed through two Pierce^TM^ High Capacity Endotoxin Removal Spin Columns (Fisher, Waltham, MA, USA, Cat# P188271) to remove the endotoxin. The endotoxin level in the final purified protein was determined to be 14 EU/mL using a Pierce^TM^ Chromogenic Endotoxin Quant Kit (Thermo Scientific, Waltham, MA, USA, Cat# A39553).

### 2.6. Vero E6 Toxin Challenging Assay

A mixture containing Vero E6 cells (1.5 × 10^4^ cells/well)**,** purified Stx2a (final 5 pg/mL or 57 fM), and diluted DARPin was seeded in 96-well plates (200 μL/well). The plates were incubated at 37 °C/5% CO_2_ for 72 h, and the cell viability was quantified using the CellTiterGlo reagent (Promega). Relative viability is calculated using the equation below:

$$Relative\;Viability=\frac{R_{\left(cells+DARPin+toxin\right)}-R_{\left(toxin\;only\right)}\;}{R_{\left(cells\;only\right)}-R_{\left(toxin\;only\right)}\;}$$

### 2.7. In Vitro Catalytic Activity Assay

Low-profile PCR tubes (200 μL capacity) were first blocked with 150 μL PBS+0.2% BSA at room temperature for 1 h and then washed twice with DPBS (Fisher, Cat# SH30028FS). Purified Stx2a-A1 (37.5 μM in DPBS) was incubated with an equal volume of DARPin (150 μM in DPBS) or DPBS alone at 37 °C for 20 min to allow DARPin to form complexes with the toxin. Next, 1.6 μL of this mixture was added to 1× NEBExpress Cell-free *E. coli* Protein Synthesis System (NEB, Cat# E5360S) containing 3 μL of S30 extract, 6 μL of Protein Synthesis Buffer, 0.2 μL T7 RNA polymerase, and 0.2 μL RNase Inhibitor, Murine. The reaction was incubated at 37 °C for 30 min, during which time the toxin Stx2a-A1 is able to inactivate the ribosomes. Next, a plasmid DNA encoding a reporter eGFP gene under a T7 promoter (75 ng) was added to the reaction to initiate RNA synthesis and protein translation. The reaction was carried out at 37 °C for 6 h, followed by overnight incubation at 4 °C to ensure complete folding of eGFP. A total of 45 μL of ddH_2_O was then added to each tube, and the mixture was transferred to a black 384-well plate (25 μL/well) for the quantification of fluorescence intensity using a Cytation 5 plate reader (Agilent). The positive controls contained no toxin, and the negative controls contained toxin, but no DARPin.

### 2.8. ELISA

The ELISA experiments were carried out essentially as described previously [40], except that Stx2a, Stx2a-A1, or Stx2a-B were used to coat the wells of the MaxiSorp immunoplates (Fisher, Nunc MaxiSorp ELISA plates, Cat#50-712-278) at 4 µg/mL. All DARPins contain a Myc tag at the N-terminus. For affinity measurement, well-bound DARPin the molecules were detected using mouse anti-c-Myc antibody (Invitrogen Cat# 13-2500) and horseradish peroxidase (HRP)-conjugated goat anti-mouse antibody (Jackson Immuno Research, Cat# 115-035-146).

### 2.9. Affinity Measurement

The affinity measurement was performed on a Fortebio BLItz instrument (Sartorius, Goettingen, Germany) using the Streptavidin sensors (Sartorius #18-5019). Purified holotoxin Stx2a (final 1 mg/mL) was first biotinylated using EZ-Link Sulfo-NHS-LC-Biotin (Pierce) at 1:10 molar ratio in PBS at 4 °C for 16 h and then passed through a Zeba spin desalting column (7K, MWCO, ThermoFisher) to remove the excess biotin molecules. Biotinylated Stx2a (0.37 µM) was captured onto the sensor. PBS with 0.1% BSA (Fisher# BP9706100) was used as the buffer for protein dilution, sensor equilibration, and washing. Subsequently, the biosensors were dipped into serially diluted DARPin proteins. Every measurement run consisted of 30 s of equilibration, 60 s of Stx2a loading, 30 s of washing, 60 s of DARPin association, and 60 s of dissociation. BLItz pro 1.3 software was used for binding detection and analysis. K_D_ values were the average of at least three K_D_ values calculated using the local fitting method.

### 2.10. Cryo-EM Sample Preparation

Stx2a and #3 DARPin were mixed at a 1:1 molar ratio (with the final concentration of the complex at 800 nM) and incubated in PBS buffer at pH 7.4 for 30 min at room temperature; 3 μL of the complex were applied to C-flat 2/1 holey carbon film 300 mesh grids at 20 °C with 100% relative humidity and vitrified using a Vitrobot (Mark III, FEI Company, Hillsboro, Oregon).

### 2.11. Cryo-EM Data Collection

The complex of Stx2a and #3 DARPin was imaged under the Thermo-Fisher Talos Arctica electron microscope (Thermo-Fisher), with a field emission gun operated at 200 kV. The microscope is equipped with a Gatan K2 summit direct detection camera (Gatan, Pleasanton, CA, USA); 3500 micrographs were collected in the electron-counting mode at 1.07Å/pix. The beam intensity was adjusted to 5 e^−^/Å^2^/s on the camera. A 33-frame movie stack was collected for each picture, with 0.2 s per frame, for a total exposure time of 6.6 s.

### 2.12. Image Processing

The collected movies for the complex of Stx2a and #3 DARPin were processed by cryoSPARC [43], (Supplementary Figure S8). These stacks were aligned using Motioncorrection 2 [44] with estimated contrast transfer functions using a patch CTF estimation. A total of 1606 micrographs with strong power spectra were used to select particles using a blob picker. After generating 2D averages from particles chosen by the blob picker, the particles were selected again by the template picker, yielding 797,974 particles. These particles were subjected to 2D classification for particle cleaning, generating 251,399 particles for ab initio reconstruction and heterogenous refinement into two classes. The two reconstructed density maps are the apo-state Stx2a and the bound-state Stx2a complexes. Further refinement was performed on each of the states. The overall resolution was assessed using the gold-standard criterion of Fourier shell correlation, with a cutoff at 0.143, between 2 half-maps from 2 independent half-sets of data.

### 2.13. Model Building

The homology model of DARPin #3 generated by SWISS-MODEL [45] and the initial model for Stx2a (pdb: 1r4p) [23] were roughly docked into the density map of Stx2a and DARPin complex. The position of Stx2a was clear docked, since subunit a and subunit b can be distinguished easily. The docking of DARPin was obtained by achieving the best fitting score calculated in Chimera [46]. The fitted model was further refined in Phenix [47] to achieve better geometry. The apo state Stx2a model was refined using the apo state Stx2a density map described above. To compare the steric positions of Gb3 and DARPin, the Gb3 positions were obtained from the solved crystal structure of the StxB and Gb3 complex (pdb: 1bos) and superimposed on the refined Stx2a-DARPin structure.

The homology model of DARPin SHT was generated by SWISS-MODEL and templated on a DARPin molecule (pdb: 1mj0). The protein structures were visualized using VMD software [48].

### 2.14. Data Availability

Models are available from the Protein Data Bank, with accession number 7UJJ for the Stx2-DARPin complex; density maps are available from EM Data Bank, with accession numbers EMD-26563 and EMD-26565 for the Stx2-DARPin complex and apo state Stx2, respectively. All other relevant data will be provided upon request.

### 2.15. Stx Toxin Challenge in Mice

All animal studies followed protocols approved by the Tufts University Institutional Animal Care and Use Committee (IACUC #G2021-160). Four- to five-week-old female Swiss Webster mice (average 25 g) were inoculated intraperitoneally with either Stx2a (60 ng) mixed with PBS (100 µL), or Stx2a (60 ng) mixed with DARPin DA1-SD5 (250 µg or 125 µg) in PBS (100 µL) immediately before injection. This toxin dose corresponds to 1.25 × the minimum lethal dose (MLD), determined previously by us [49]. The mice were monitored for changes in body weight, as well as neurologic signs, including change in demeanor, activity, and responsiveness to stimuli. When the mice exhibited progressive clinical signs, became less responsive to stimuli, or lost 20% of their bodyweight, they were humanely euthanized.

## 3. Results

### 3.1. Selection of Monomeric Stx2a-Neutralizing DARPins

We first developed a strategy to purify holotoxin Stx2a. A 6xHis tag was fused to the C-terminus of the B subunit, and the holotoxin was expressed in the periplasmic region of *E. coli* and purified by one-step affinity purification. To remove all monomeric A (35 kDa) and B subunits (7 kDa), the purified protein was concentrated via ultrafiltration (MWCO 50 kDa) to recover only the assembled holotoxin (AB_5_, 70 kDa, Figure S1). The purified Stx2a holotoxin is highly toxic to Vero E6 cells, with an estimated IC_50_ of <5 pg/mL, confirming the correct folding of the holotoxin (Figure S2). Increasing the toxin concentration from 10 to 200 pg/mL resulted in moderately reduced cell viability, from ~20% to ~5%. Our assay condition, in which the toxin was added to a low density of Vero cells at the time of cell seeding, may be responsible, in part, for this seemingly flat toxicity curve.

**Figure 1:**
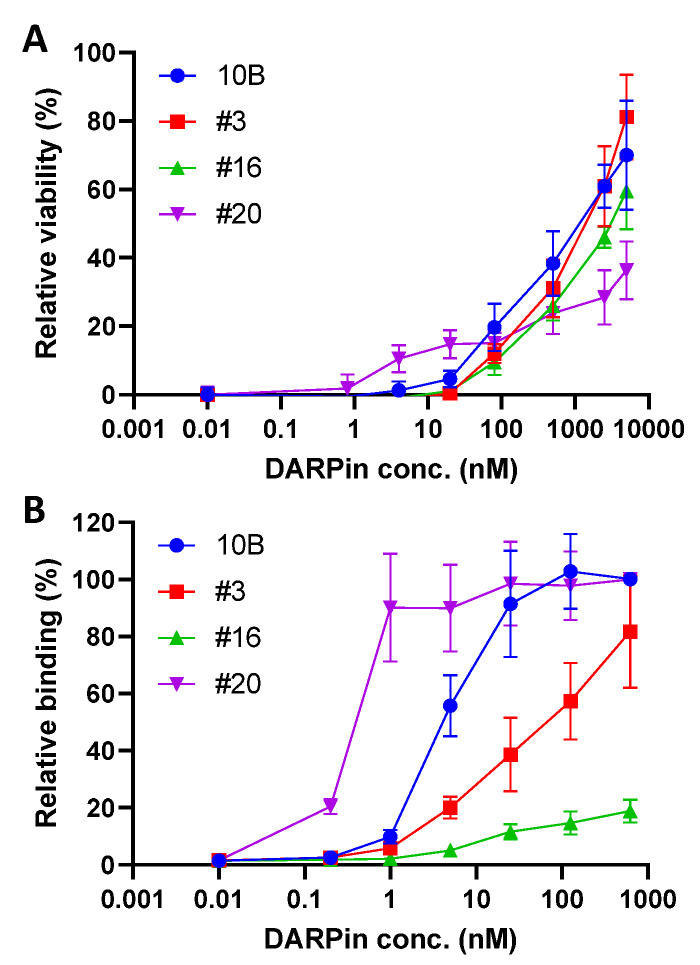
Characterization of parental anti-Stx2 DARPins discovered from a phage-displayed naïve DARPin library. (**A**) Monomeric DARPins protected Vero E6 cells from Stx2a-induced cytopathic effect. Each well contains 1.5 × 10^3^ Vero cells, 5 pg/mL (or 57 fM) Stx2a, and the appropriately diluted DARPin. Error bars represent the standard deviation of two independent experiments performed in duplicate. (**B**) Relative binding of monomeric DARPins to Stx2a, determined using ELISA. Error bars represent the standard deviation of two independent experiments.

Using an in-house DARPin library with ~2 × 10^9^ diversity [39], we carried out 4 rounds of phage panning against biotinylated holotoxin Stx2a. The enriched phage pool from the 3rd round of selection, which showed significantly enhanced ability to bind Stx2a (Figure S3), was cloned into the pET28a vector and transformed into *E. coli* BL21(DE3) cells for high-level DARPin expression. A total of 752 colonies were selected and grown in 96-well plates, and the cell lysates were subjected to functional screening for those able to rescue Vero E6 cells from Stx2a toxicity (see methods). Next, 48 candidate clones were selected and sequenced, yielding 21 unique clones. These clones were individually expressed and purified via affinity chromatography and their toxin neutralization and binding abilities were evaluated using the Vero-E6 toxin challenge assay and ELISA, respectively. Among these, 19 clones exhibited measurable toxin neutralization activity. The four best clones (#3, 10B, #16, and #20) neutralized Stx2a (5 pg/mL or 57 fM) with EC_50_ >1 μM (Figure 1A), which is likely to be inadequate for therapeutic applications. The ability of these DARPin molecules to bind Stx2a does not appear to correlate with their neutralization potency (Figure 1B). We further investigated the subunits targeted by these DARPins using purified A1- and B subunit of Stx2a (Figure S1). DARPin 10B and #20 bind strongly to the A1 subunit, while DARPin #3 and #16 failed to bind either of the subunits individually, despite their apparent abilities to bind the holotoxin (Figure S4), indicating that these two DARPins may target a conformational epitope(s) present only on the AB_5_ holotoxin.

### 3.2. Systematic Affinity Maturation

**Figure 2:**
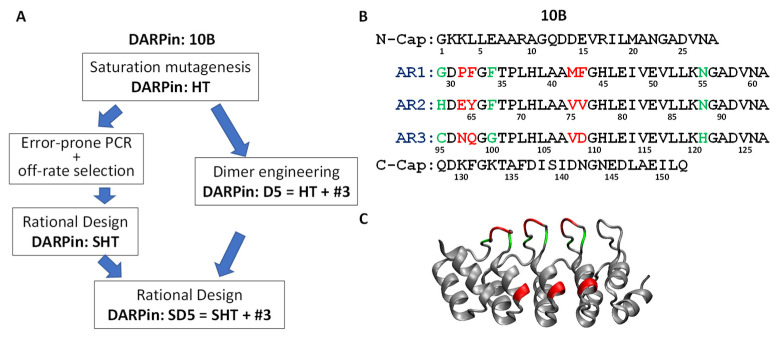
(**A**) Schematic of the affinity maturation strategy. Sequence (**B**) and homology model (**C**) of DARPin 10B. Both green and red residues were randomized in the original DARPin library, and the red residues were subjected to saturation mutagenesis.

The high toxin neutralization activity, indicating the association of an important epitope on the toxin, and the medium toxin binding affinity of DARPin 10B caused it to be selected for affinity maturation. An overview of our systematic affinity maturation strategy is shown in Figure 2A. We decided to first perform saturation mutagenesis of the residues in direct contact with the toxin. To maximize the diversity while maintaining a manageable library size, six saturation mutagenesis libraries were constructed, with each library consisting of a consecutive dipeptide randomized to all 20 amino acids (Figure 2B, red residues). A total of 4800 colonies (800 per library) were screened using the Vero E6 toxin challenge assay, yielding 30 candidates with improved toxin-neutralization activity. These candidates were sequenced, revealing 19 unique clones. Interestingly, all these clones contain mutations at positions 108–109. The best DARPins, HT and RI (denoting the amino acids at positions 108, 109), showed ~4-fold improved toxin neutralization activity in the Vero E6 toxin challenge assay and ~8-fold improved toxin-binding ability in ELISA (Figure S5).

To further improve the toxin-neutralization potency through an avidity effect, we carried out a dimer library screening in which the 19 different 1st-generation DARPin monomers, plus HT and RI, were randomly linked together via a flexible (G_4_S)_4_ linker (expected to span ~55 Å). The theoretical library size is 441 (21 × 21). A total of 768 individual clones were screened using the Vero-E6 toxin challenge assay with 5 pg/mL Stx2a, and 20 candidates with enhanced neutralization activity were identified. The sequencing of these clones revealed 10 unique combinations. These dimers were purified by one-step metal chelation chromatography, and their toxin neutralization activity was evaluated. All dimers exhibit significantly enhanced neutralization potency compared to the best monomer HT (Figure S6). The best dimer—D5—is composed of DARPin #3 and HT, and it neutralized Stx2a with EC_50_ of 38.3 nM.

Concurrently, we sought to further reduce the off-rate of the monomeric DARPins (Figure 2A). For therapeutic applications, a critical factor limiting the efficacy of a binding protein (e.g., antibody) is its retention time on the target [50]. The selection of variants with a slower off-rate using competitive off-rate selection has proven to be a powerful method for improving affinity [41,42]. Error-prone PCR was used to generate randomized libraries of DARPin monomers. After three rounds of off-rate selection, a 4th round of phage panning was carried out using biotinylated Stx2a (2 nM) under standard conditions.

**Figure 3:**
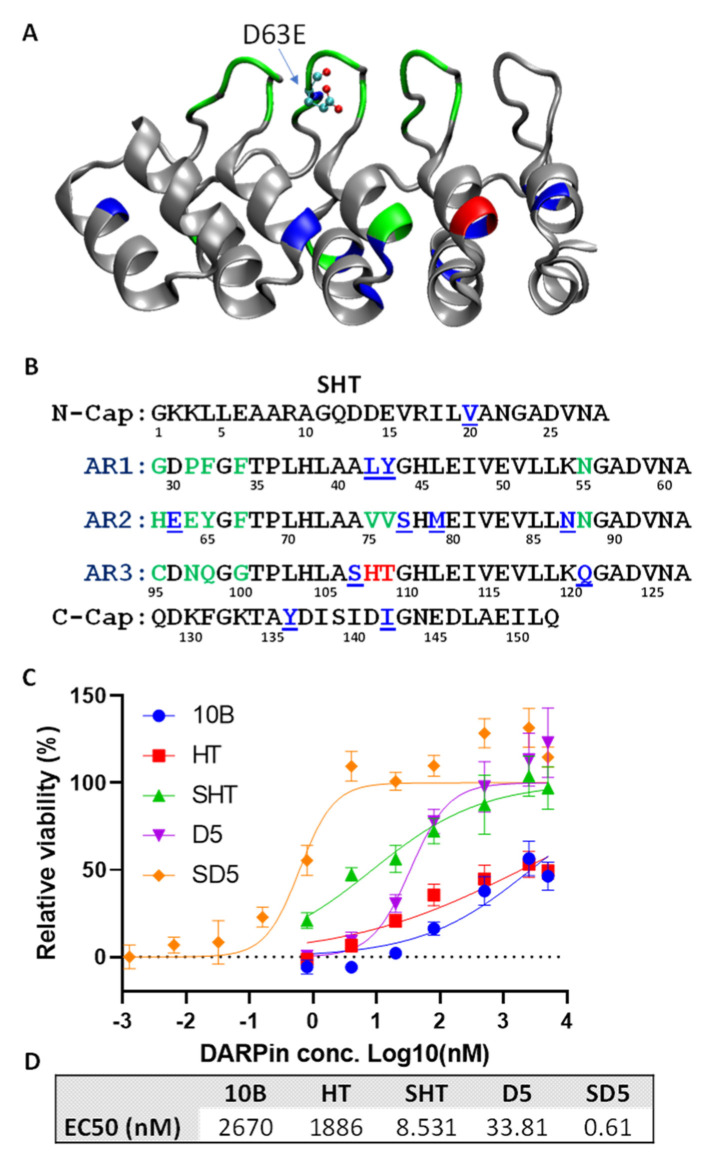
Homology model (**A**) and amino acid sequence (**B**) of DARPin SHT showing the derivation of key putative Stx2a-binding residues. The green, blue, and red residues are derived from the original DARPin library, error-prone PCR library, and saturation mutagenesis library. (**C**) Protection of Vero E6 cells by the different DARPins. Vero E6 cells (1.5 × 10^3^ cells/well) were incubated with 5 pg/mL (57 fM) Stx2a and the appropriately diluted DARPin for 3 days. Error bars represent the standard deviation of two independent experiments performed in duplicate. (**D**) Summary of EC_50_ values.

The enriched 4th-round selected error-prone PCR phage pool was cloned into pET28a for DARPin expression, and the *E. coli* lysates were subjected to screening for Stx2a binding. A total of 400 colonies were screened, yielding 28 unique clones with enhanced Stx2a binding relative to clone HT. Remarkably, all of these clones contained the D63E mutation (Figure S7), pointing to an important role of this residue in Stx2a binding. Mutation D63E is located on AR2 near the tip of a binding loop (Figure 3A) and is sandwiched between target binding residues at position 62, 64, and 65 that are randomized in the original DARPin library (Figure 3B). Residue at position 63 was believed not to be directly involved in target binding. Although both Asp and Glu are negatively charged, the side chain of glutamic acid has one additional carbon bond and may thus enable the formation of a more favorable interaction with the target protein. A total of 21 clones are derived from DARPin HT, and 7 are from DARPin RI, likely due to the stronger neutralization activity of these parental DARPins (Figure S7). Point mutations from variants derived from DARPin HT were rationally combined, based on their abundance and proximity to the target binding residues to generate variant SHT, with a total of 11 mutations compared to the parent clone HT (Figure 3B). DARPin SHT exhibits a >100-fold enhanced Stx2a neutralization potency (EC_50_ 8.5 nM) relative to HT (EC_50_ 1.9 μM) (Figure 3D).

Finally, the HT in the best dimer DARPin D5 was replaced with SHT to give rise to dimer SD5 (#3-SHT) (Figure 2A). DARPin SD5 neutralized Stx2a with EC_50_ of 0.6 nM (Figure 3D), which is 4450-fold more potent than the best 1st generation DARPin 10B (EC_50_ 2.7 µM), which is a strong testament to the power of the sequential protein engineering approach employed in this study. We also determined the potency of SD5 against Stx2c, another genotype commonly involved in HUS. Despite high sequence homology (98.7% identical), SD5 showed slightly reduced, albeit still potent, antitoxin activity toward Stx2c with EC_50_ of 7.3 nM (Figure S8).

### 3.3. In Vitro Characterization of DARPin Molecules

Since DARPin 10B binds the A subunit of Stx2a, we first evaluated its ability to inhibit the toxin’s catalytic activity. In the cell, the A subunit is proteolytically processed to form the catalytically active A1 fragment and the smaller A2 fragment that remains associated with the pentameric B subunit [51]. The A1 fragment is recognized by the ER export mechanism and translocated to the cytosol [52], where it inhibits protein synthesis by removing an adenosine from the 3′ region of the 28S rRNA, triggering apoptosis [53,54].

**Figure 4:**
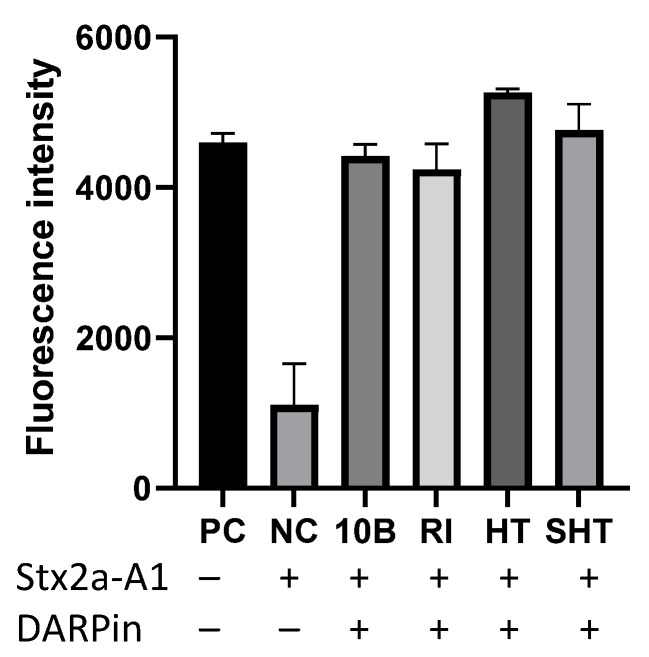
DARPin molecules protect ribosomes from intoxication by Stx2a-A1 in vitro. Purified Stx2a-A1 (2.5 μM) was incubated with DARPin molecules (final 10 µM) in PBS supplemented with BSA (40 mg/mL) for 30 min at 37 °C before being added to the 1x NEBExpress Cell-free *E. coli* protein synthesis system, which contains both the translation and transcription machinery. The mixture was incubated at 37 °C for 30 min before the addition of a reporter plasmid encoding GFP, and the protein synthesis was continued at 37 °C for 6 h, followed by overnight incubation at 4 °C to ensure complete folding of GFP. The amount of GFP produced was quantified using a fluorescent plate reader and compared to that obtained from reactions lacking any toxin (positive control, PC) and reactions with toxin, but without any DARPin (negative control, NC).

We constructed a Stx2a-A1 fragment with a C-terminus 6xHis tag, purified the protein by metal chelation chromatography (Figure S1), and determined the ability of DARPins to rescue ribosomes from intoxication by Stx2a-A1. As shown in Figure 4, Stx2-A1 efficiently inhibited the protein synthesis, which can be rescued by DARPin 10B and its progenies. However, DARPin HT and SHT did not exhibit greater potency than 10B. This may be due to the relatively high concentration of DARPin (i.e., 10 µM) used in this study, which may obscure the differences in target binding affinity between these DARPins.

**Figure 5:**
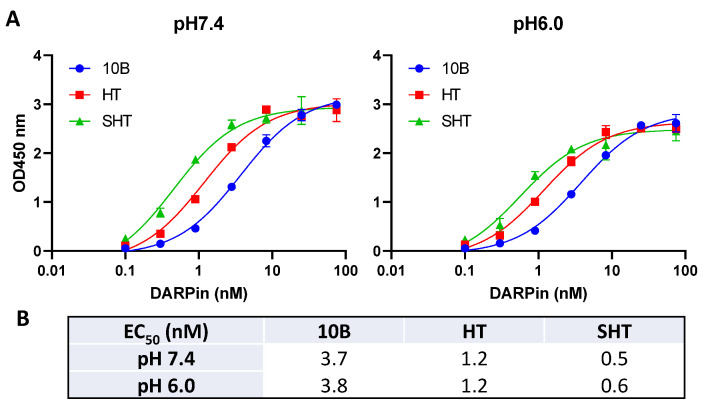
Relative binding of DARPins to Stx2a-A1 fragment determined by ELISA. (**A**) DARPins were added to MaxiSorp plates coated with 4 µg/mL Stx2a-A1. A representative experiment with the mean of 2 technical replicates is shown. (**B**) Summary of the calculated EC_50_ values.

We next compared the target binding ability of these DARPins using immobilized Stx2a-A1 by ELISA at both neutral (pH 7.4) and acidic (pH 6.0) conditions to mimic the environment in the extracellular space and within the endosome upon toxin internalization, respectively. The target binding ability gradually improved in the course of the engineering with DARPin SHT, exhibiting 6-7-fold increased binding ability toward immobilized Stx2a-A1 over SHT (Figure 5). In addition, as anticipated, the binding interaction between the DARPins and Stx2a-A1 appeared to saturate at concentrations >100 nM, consistent with the observed similar inhibitory activity in the above in vitro catalytic assay.

**Figure 6:**
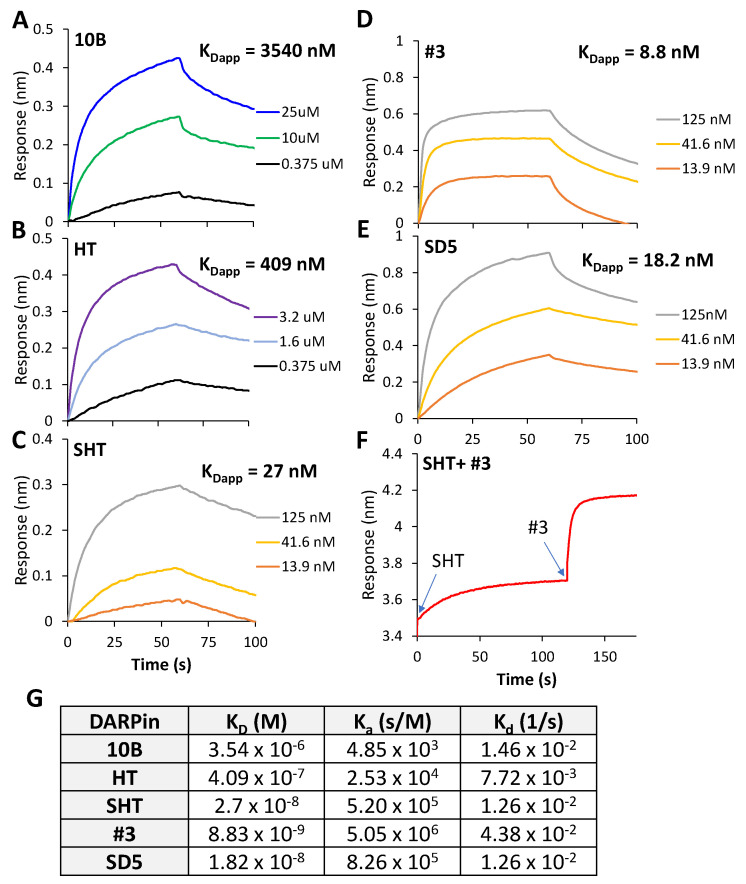
Binding kinetics of selected DARPins. (**A**–**E**) Sensorgrams of the various DARPins under equilibrium binding condition. (**F**) Sensorgram of a sequential binding experiment in which the biosensor was first loaded with DARPin SHT (125 nM) for 120 s to reach saturation. The sensor was then dipped into a buffer containing DARPin #3 (125 nM) for 30 s to record the binding kinetics. (**G**) Summary of the binding and rate constants.

We also compared the affinity of the different DARPin molecules to Stx2a holotoxin using bio-layer interferometry (BLI, Figure 6). Remarkably, the affinity of SHT (K_Dapp_ = 27 nM) is 128-fold stronger than that of 10B (K_Dapp_ = 3450 nM). The affinity of HT (K_Dapp_ = 409 nM) is in between that of 10B and SHT. Although the trend of affinity improvement from 10B to SHT is the same in ELISA and BLI, the fold difference is drastically different. Most likely, there is a slight conformational difference between Stx2a-A1 (used in the ELISA) and Stx2a holotoxin (used in BLI), caused by either the protein tertiary structure or the immobilization condition. In addition, unmodified Stx2a-A1 was used to coat the ELISA plate directly, while Stx2a with biotin molecules conjugated to random surface lysin residues were used in the BLI experiment.

Since the other constituent of our best DARPin dimer SD5 is DARPin #3, we also determined the affinity of DARPin #3 and SD5 (Figure 6D,E). DARPin #3 binds Stx2a holotoxin with a slightly higher affinity (K_Dapp_ 8.8 nM) than SHT. Interestingly, the affinity of dimer SD5 (K_Dapp_ = 18.2 nM) falls in between its constituent DARPins. This is somewhat unexpected, since we anticipated the dimer to exhibit even higher affinity through the avidity effect [39]. The weaker affinity of SD5 may reflect some intramolecular hindrance between #3 and SHT, or a reorientation of SD5 upon binding to Stx2a.

To confirm that DARPin #3 and SHT can bind simultaneously to the same Stx2a molecule, a sequential binding experiment was carried out (Figure 6F). An Stx2a-coated sensor was loaded first with SHT at a high concentration (i.e., 125 nM) to occupy all the binding sites on Stx2a before the loading of DARPin #3. The binding of SHT did not impede the binding of #3, indicating that SHT and #3 bind non-competitively to Stx2a.

### 3.4. In Vivo Efficacy of DARPin

**Figure 7:**
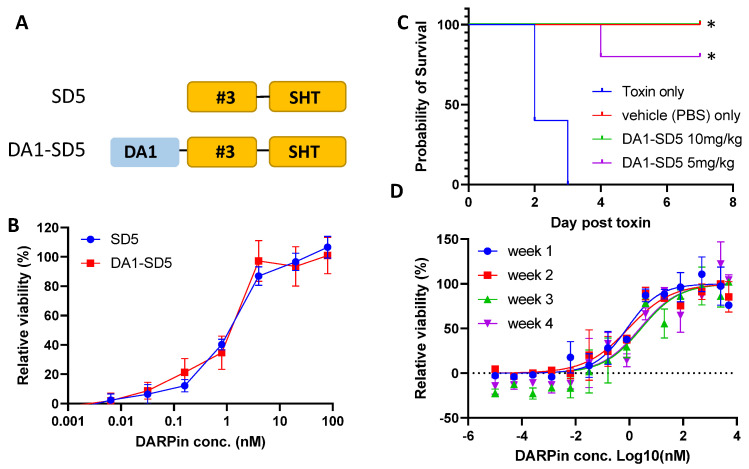
Evaluation of the in vivo activity. (**A**) Schematic of DARPin DA1-SD5 in comparison to SD5. (**B**) DARPin DA1-SD5 retains similar toxin-neutralization potency as SD5, as determined by Vero E6 cell viability assay. (**C**) DARPin DA1-SD5 protected mice from Stx2a toxicity (*n* = 5); Mantel–Cox test, * *p* < 0.002. (**D**) Toxin neutralization activity of DA1-SD5 after storage at room temperature for 1–4 weeks, as determined by Vero E6 cell viability assay.

Due to its small size, 90% of monomer DARPin is excreted within 7.5 min in mice [55]. Piggybacking onto albumin proteins has been shown to be an effective strategy to increase the half-life of therapeutic proteins by taking advantage of the long circulation half-life of serum albumin protein. Previously, a serum albumin-binding DARPin was engineered and found to significantly improve the circulation half-life of the fusion protein to 27–80 h in mice and 2.6–20 days in cynomolgus monkeys [38]. To extend the in vivo half-life, DARPin SD5 was fused to an albumin-binding DARPin DA1 [38] (Figure 7A). The resulting DARPin DA1-SD5 exhibits identical Stx2a neutralization activity to the parent SD5 (Figure 7B), indicating that DA1 fusion does not interfere with the SD5-toxin interaction.

To evaluate the in vivo efficacy, Swiss Webster mice (group of 5) were challenged intraperitoneally with either Stx2a (60 ng per mouse) alone, or with a mixture of Stx2a (60 ng) and DA1-SD5 (5 or 10 mg/kg) on day 0 (Figure 7C). Mice were closely monitored after toxin challenge and were humanely euthanized when they exhibited neurologic signs, became less responsive to stimuli, or lost 20% of their body weight. All mice receiving the toxin alone died within 3 days. On the other hand, all mice receiving the same dose of Stx2a plus 10 mg/kg of DA1-SD5, and 4 out of the 5 mice receiving the toxin and 5 mg/kg of DA1-SD5, survived until the end of the study, with no clinical signs of disease. These data offer an encouraging assessment of the in vivo efficacy of our engineered DA1-SD5.

Finally, we assessed the storage stability of DA1-SD5 in PBS. After 4 weeks of storage at room temperature, negligible reduction in neutralization activity was observed (Figure 7D), confirming the excellent shelf life of DARPin molecules. This is not surprising, as DARPins were previously shown to exhibit superior storage stability, with a mere 0.2% loss of activity over 18 months at 5 °C and 2.4% activity loss over 6 months at 25 °C [36].

### 3.5. Cryo-EM Study of DARPin #3 and Stx2a

Since DARPin #3 failed to bind either the Stx2a-A1 or Stx2a-B subunits in ELISA (Figure S4), cryo-EM was used to elucidate its epitope on the Stx2a holotoxin. Reference-free 2D classification revealed multiple classes of Stx2a (Figure S9A). Some classes lack any DARPin density and correspond to the apo state of Stx2a, while others clearly contain extra density that can only be attributed to the bound DARPin molecule (Figure S9B). Heterogenous refinement confirmed that there are two particle groups: apo-state Stx2a and Stx2a-DARPin complex (Figure S10). Both of the maps reach ~6 Å, which allow us to dock the rigid domains of Stx2a (PDB: 1r4p), as well as DARPin, into the density maps.

**Figure 8:**
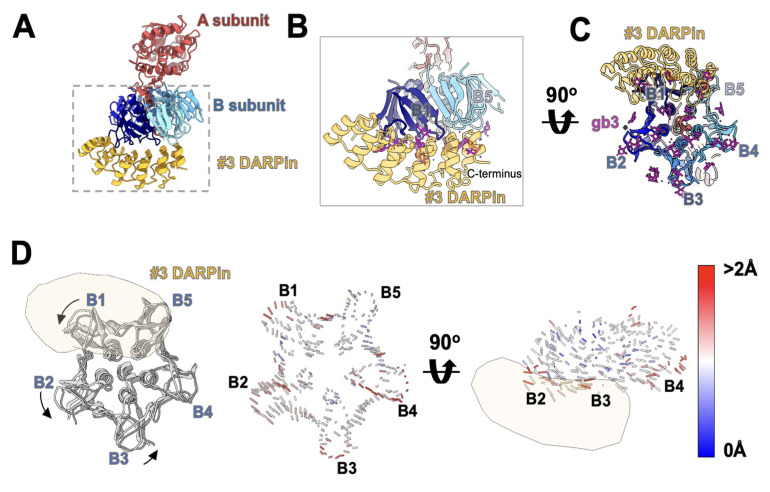
Cryo-EM structure of the Stx2a-DARPin #3 complex. (**A**) Side view of the complex. A subunit is colored red. The individual monomers in the B subunit are colored in different shades of blue. DARPin#3 is colored orange. (**B**) A zoomed-in view of the complex. The bound modeled Gb_3_ molecules are colored magenta. (**C**) Bottom view of the complex. (**D**) Analysis of B subunit rotation in the apo and bound states. The vectors represent the relative displacement between the equivalent Ca atoms in the B subunit in the apo- and DARPin-bound state, with large and small movements indicated by red and blue colors, respectively.

Based on the EM structure, DARPin #3 binds unequivocally to the basal side of Stx2a and interacts with two of the monomers in the B subunit pentamer (Figure 8A and Figure S9B). Comparing to the apo state of Stx2a, the DARPin-bound Stx2a undergoes a conformational change in the B subunit (Supplemental Video S1). After aligning the corresponding A subunits between the apo state and the DARPin-bound state, a significant movement between the respective B subunits was observed. The B subunit closest to the C-terminus of DARPin #3 is defined as B1, while the remaining B subunits were named sequentially in a counterclockwise direction (Figure 8C). The association of DARPin #3 induced a counterclockwise rotation of subunit B1, B2, and B3 (Figure 8D left), and an upward movement of subunit B4 (Figure 8D, right), thus distorting the five-fold symmetry of the pentameric B subunit. The basal side of the B subunit is responsible for binding the carbohydrate moiety of the glycosphingolipid Gb_3_ on the cell surface. DARPin #3 blocks the interaction pockets of monomer B1 and B5, thus neutralizing the toxin.

## 4. Discussion

Shiga toxin (Stx)-producing *E. coli* bacteria (STEC), a common contaminant of meats, spinach, bean sprouts, and other foods, causes both outbreaks and sporadic cases of bloody diarrhea around the world, resulting in significant morbidity [56,57,58]. In some patients, mostly young children and the elderly, infections by STEC are often accompanied by the more serious hemolytic uremic syndrome (HUS, up to 25% [59]), a condition characterized by thrombocytopenia, hemolytic anemia, and kidney failure [60,61], with a mortality rate of 5−10% [62].

Using directed evolution combined with rational design, we engineered a panel of DARPin molecules with potent Stx2a-neutralization activity. The best DARPin, SD5, neutralized Stx2a with an EC_50_ of 0.6 nM (Figure 3) with a K_d_ of 18 nM (Figure 6), and effectively protected mice from Stx2a intoxication in the mouse toxin challenge model (Figure 7). The constituents of DARPin SD5 are monomer DARPins #3 and SHT. DARPin SHT is derived from DARPin 10B, which binds the Stx2a A subunit (Figure S4) and inhibits the enzymatic activity of the toxin (Figure 4). This mode of action is similar to that of antibody 11E10 [63] and 5C12 [33]. The toxin-neutralization potency of DARPin SHT is >300-fold and >220-fold stronger than its grandparent DARPin 10B and parent DARPin HT, respectively (Figure 3D). Among the 11 mutations acquired by SHT, only 2 (108–109) are directly engaged in target binding. Residues 108–109 are located on the a-helix in AR3 and were originally occupied by Val-Asp in DARPin 10B. Through saturation mutagenesis, we obtained DARPin HT and RI, whose residues at these positions were mutated to His-Thr and Arg-Ile, respectively. Both HT and RI showed similarly improved binding affinity toward Stx2a (Figure S5B), indicating an important role of these residues in target interaction. However, the replacement of hydrophobic Val and negatively charged Asp in DARPin 10B with nucleophilic Thr and positively charged His in HT, and negatively charged Arg and hydrophobic Iso in RI, is not anticipated, and their contribution is unclear without further structural studies. The remaining nine mutations in SHT are believed not to directly participate in target interaction, but rather to subtly influence the binding interface. These mutations further increased the toxin neutralization potency of SHT by >220-fold compared to HT. The increased affinity of SHT over HT is derived by both a faster K_a_ and a slower K_d_ (Figure 6G), showing the success of our off-rate selection.

Among all the mutations introduced by error-prone PCR, D63E was present in all the mutants and was the most notable. Asp63 is located on a binding loop on AR2 and is sandwiched between His62 and Glu64, both of which are deemed important for target binding and are randomized in the original DARPin library [64]. However, position 63 is believed not to participate in target binding and is occupied by Asp in the parental DARPin structure. Among all the natural ankyrin repeat proteins, this position is highly conserved and is usually occupied by Asp, Thr, or Ala [65]. Although the exact mode of action of D63E is unclear without additional structural information, the increased affinity contributed by this mutation points to an important role of this residue in target interaction.

The other constituent of SD5—DARPin #3—failed to bind significantly to both the purified A and B subunit alone in ELISA, despite its high affinity to the full-length toxin (Figure S4). This apparent conundrum was later resolved by cryo-EM structural study, which revealed that DARPin #3 unequivocally binds the B subunit at the basal surface of Stx2a (Figure 8). The association of DARPin #3 induces a conformational change in the B subunit (Movie 1) that distorts its five-fold symmetry. Such a conformational change may not be possible in the purified Stx2a-B subunit lacking the A2 subunit. At the resolution available, we are unable to discern whether DARPin #3 also interacts with the A2 tail which protrudes from the B subunit. Since the B subunit adopts a five-fold symmetry, the fact that only one DARPin molecule was seen in association with the B subunit may indicate the involvement of the A2 domain. This is in contrast to a previously developed Stx2e-neutralizaing nanobody, NbStx2e1, which associates with the B subunit in such a way that five molecules are able to bind simultaneously to Stx2e without any steric hindrance [66]. The ability of DARPin #3 to distort the tertiary structure of the B subunit is unprecedented and significant, and may derive from the structural rigidity of DARPins [35] and an underappreciated structural flexibility of the toxin B subunit.

Finally, using a mouse toxin challenge model, DARPin DA1-SD5 was shown to significantly protect mice from Stx2a intoxication (Figure 7C). In our study, DARPin was pre-mixed with the toxin before injection. This is very different from the event of STEC infection, in which toxins are gradually released from the bacteria within the GI tract and translocated to the blood stream. Efforts to evaluate our DARPin in an infection model are currently underway. Furthermore, DA1-SD5 exhibited excellent storage stability, with virtually no activity loss after storage at room temperature for 4 weeks (Figure 7D). This, combined with its ease of expression (up to 200 mg protein per liter of shaker flask *E. coli* culture [35] and 15/L in *E. coli* high cell-density fermentation [36]), renders the anti-toxin DARPin highly attractive for clinical development. Although the toxin challenge model used here does not completely recapitulate STEC infection during which the toxin is secreted by the bacteria residing within the gut lumen, from where the toxin travels to the kidney upon translocation to cause renal damage, systemically delivered Stx2a induces a similar renal damage phenotype [49] as those observed during infection [67,68]. This mouse toxin challenge model has been extensively used in the preclinical evaluation of anti-toxins against Stx2 [49] and has yielded outcomes consistent with the piglet infection model [33]. Therefore, we believe that our in vivo study provides a fair assessment of the DARPin’s therapeutic potential.

In summary, we report the engineering of a panel of DARPin molecules with potent Stx2a-neutralization activity. Our best DARPin, DA1-SD5, potently protects mice from Stx2a toxicity, exhibits excellent long-term stability at room temperature, and has the potential to be manufactured at relatively low cost, rendering it an attractive candidate for further clinical development. In addition, our protein engineering approach, combining directed evolution with rational design, offers an effective method for engineering other binder proteins.

## 5. Patents

A provisional patent on the engineered DARPins has been submitted.

## Data Availability

Models are available from the Protein Data Bank, with accession number 7UJJ for the Stx2-DARPin complex; density maps are available from EM Data Bank, with accession numbers EMD-26563 and EMD-26565 for the Stx2-DARPin complex and apo state Stx2, respectively. All other relevant data will be provided upon request.

## Supplementary Materials

The following supporting information can be downloaded at: https://www.mdpi.com/article/10.3390/bioengineering9100511/s1, Figure S1: SDS image of the purified proteins and their amino acid sequences; Figure S2: Toxicity of holotoxin Stx2a; Figure S3: Enrichment of Stx2-binding DARPins after phage panning; Figure S4: ELISA-based assay to evaluate the ability of monomeric DARPins to bind the A- and B-subunits of Stx2a; Figure S5: Comparison of DARPins HT, RI and 10B; Figure S6: Activity comparison of DARPin dimers; Figure S7: Sequence alignment of off-rate mutants; Figure S8: Comparison of Stx2a and Stx2c; Figure S9: Cryo-EM analysis of Stx2a and DARPin #3 complex; Figure S10: Cryo-EM Data processing; Video S1: Complex of DARPin #3 and Stx2a.
