# Supplementary material for: A Multi-Specific DARPin Potently Neutralizes Shiga Toxin 2 via Simultaneous Modulation of Both Toxin Subunits
Source: Bioengineering (Basel). 2022 Sep 27;9(10):511. doi: 10.3390/bioengineering9100511 (PMC9598796; doi:10.3390/bioengineering9100511)
Supplement: Supplementary file 1 [file bioengineering-09-00511-s001.zip › 3_Supplementary Information ZC090822_accept changes.pdf]

**Supplementary Information**

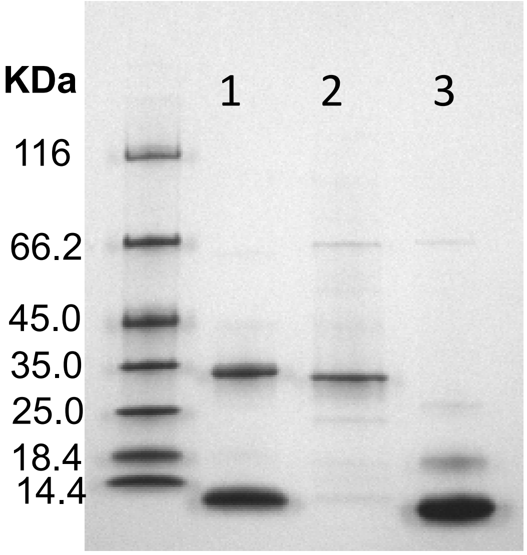


1. Stx2a holotoxin
2. Stx2a-A1 subunit
3. Stx2a B subunit

**Amino acid sequences:**

**Stx2a holotoxin:**

MKCILFKWVLCLLLGFSSVSYSREFTIDFSTQQSYVSSLNSIRTEISTPLEHISQGTTSVSVINHTPPGSYFAVDIRGLDVYQARFDHLR
LIIEQNNLYVAGFVNTATNTFYRFSDFTHISVPGVTTVSMTTDSSYTTLQRVAALERSGMQISRHSLVSSYLALMEFSGNTMTRDA
SRAVLRFVTVTAEALRFRQIQREFRQALSETAPVYTMTPGDVDLTLNWGRISNVLPEYRGEDGVRVGRISFNNISAILGTVAVILNC
HHQGARSVRAVNEESQPECQITGDRPVIKINNTLWESNTAAAFLNRKSQFLYTTGK

taaaggagttaagc

MKKMFMAVLFALASVNAMAADCAKGKIEFSKYNEDDTFTVKVDGKEYWTSRWNLQPLLQSAQLTGMTVTIKSSTCESGSGFAE
VQFNNDLEHHHHHH

DNA sequence sandwiched between the A and B subunit
Signal peptide

**Stx2a-A1:**
MKCILFKWVLCLLLGFSSVSYSREFTIDFSTQQSYVSSLNSIRTEISTPLEHISQGTTSVSVINHTPPGSYFAVDIRGLDVYQARFDHLR
LIIEQNNLYVAGFVNTATNTFYRFSDFTHISVPGVTTVSMTTDSSYTTLQRVAALERSGMQISRHSLVSSYLALMEFSGNTMTRDA
SRAVLRFVTVTAEALRFRQIQREFRQALSETAPVYTMTPGDVDLTLNWGRISNVLPEYRGEDGVRVGRISFNNISAILGTVAVILNC
HHQGARSVRAVNEESQPEGGSLEHHHHHH

**Stx2a-B:**

MKKMFMAVLFALASVNAMAADCAKGKIEFSKYNEDDTFTVKVDGKEYWTSRWNLQPLLQSAQLTGMTVTIKSSTCESGSGFAE
VQFNNDHHHHHH

**Figure S1. An SDS-PAGE (4-15%) image of the purified proteins under reducing conditions and their protein sequences.**

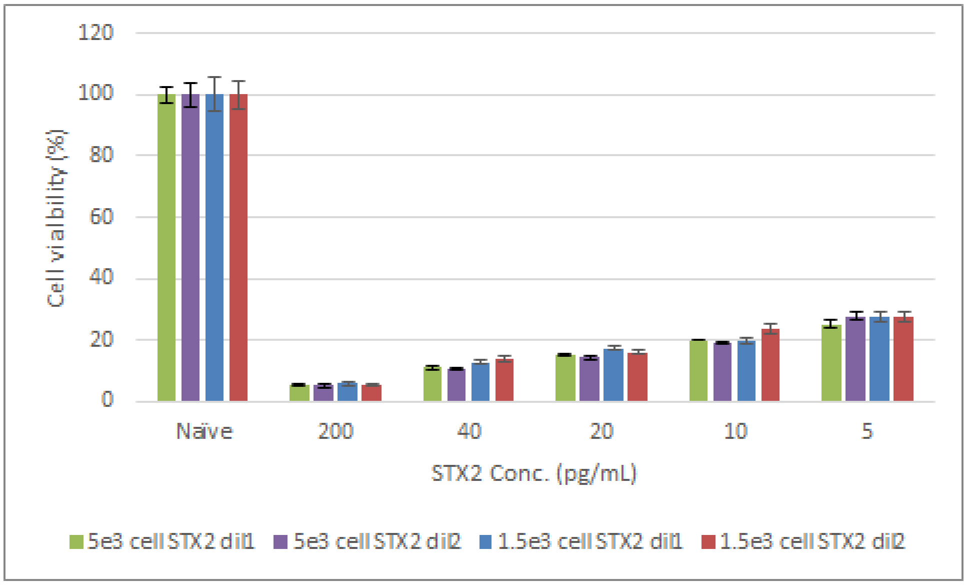


**Figure S2. Toxicity of holotoxin Stx2a.** Vero E6 cells ($1.5 \times 10^3$ cells/well) were mixed with the appropriately diluted Stx2a and the mixture was added to 96-well plate. The cell viability was quantified 3 days later using the CellTiterGlo assay.

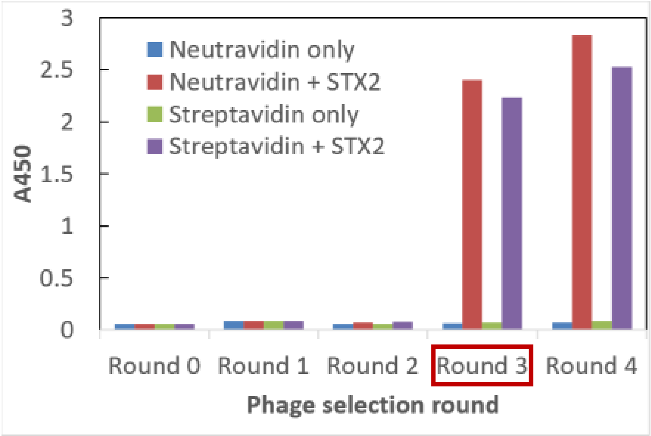


Cloned into pET28a and transformed BL21(DE3) cells

**752 clones** were picked and DARPin expressions were induced by IPTG (0.5 mM)

**48 clones** were chosen, DARPins were expressed in AI medium, and ELISA and neutralization assay were performed

**Figure S3. Enrichment of STX2-binding DARPins after phage panning.** Successive rounds of a DARPin-phage library were panned against STX2 using streptavidin-coated magnetic beads coated with 100 nM biotinylated-STX2 and MaxiSorp 96-well plates coated with neutravidin (66 nM) and 100 nM biotin-STX2 in alternative rounds. The binding of phage recovered from successive rounds of panning to STX2 was quantified using ELISA. Significant binding was observed after three rounds of panning.

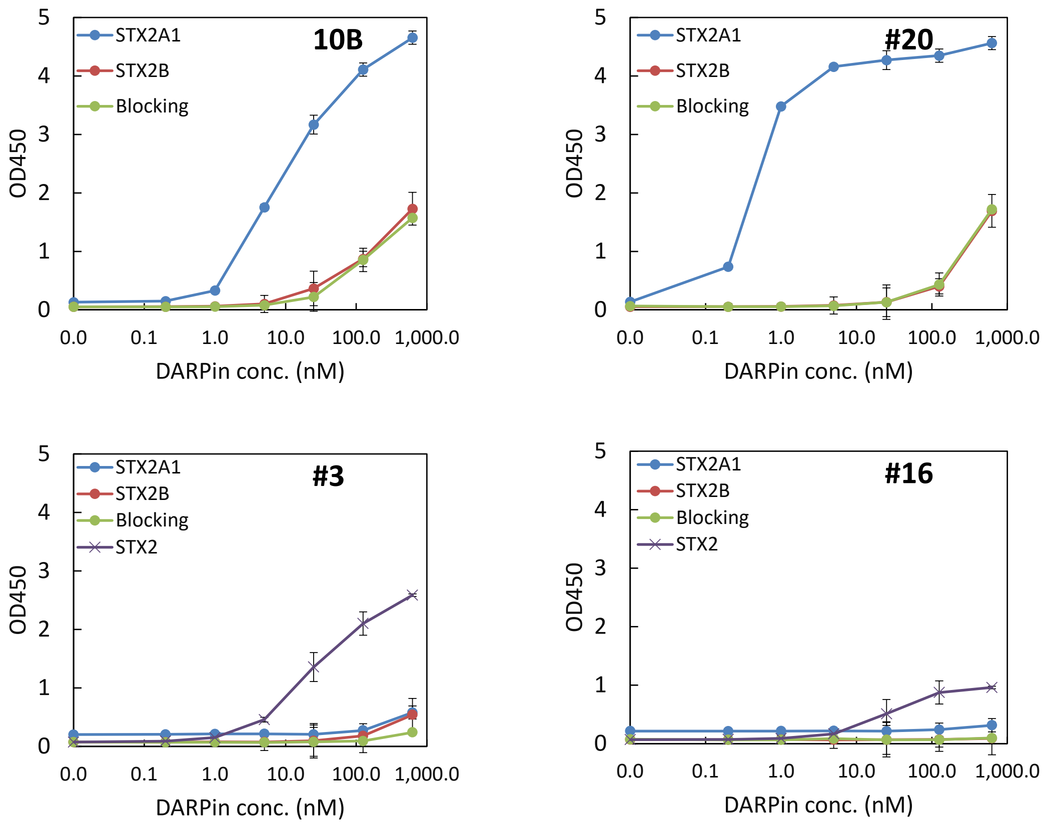


**Figure S4.** ELISA-based assay to evaluate the ability of monomeric DARPins to bind the A- and B-subunits of Stx2a. The error bars represent the mean deviation of duplicate samples from one representative experiment.

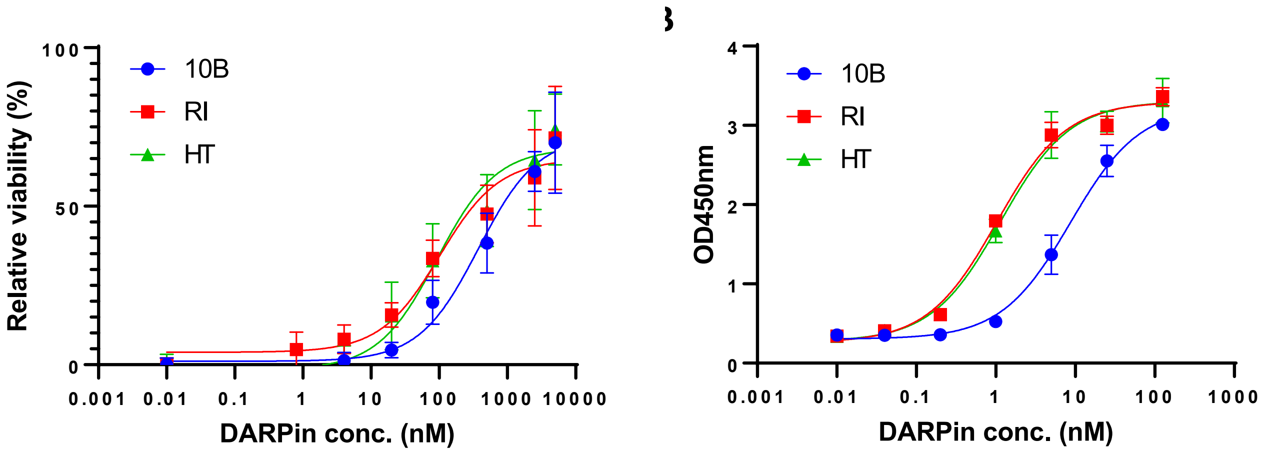


**HT:**

GKKLLEAARAGQDDEVRILMANGADVNA/GDPFGFTPLHLAAMFGHLEIVEVLLKNGADVNA/HDEYGFTPLHLAA VVGHLEIVEVLLKNGADVNA/CDNQGGTPLHLAAHTGHLEIVEVLLKHGADVNA/QDKFGKTAFDISIDNGNEDLA EILQ

**RI:**

GKKLLEAARAGQDDEVRILMANGADVNA/GDPFGFTPLHLAAMFGHLEIVEVLLKNGADVNA/HDEYGFTPLHLAA VVGHLEIVEVLLKNGADVNA/CDNQGGTPLHLAARIGHLEIVEVLLKHGADVNA/QDKFGKTAFDISIDNGNEDLA EILQ

**Figure S5.** Relative viability **(A)** and binding ability **(B)** of DARPin HT, RI and 10B. **(C)** Amino acid sequences of DARPin HT and RI. The ELISA plates were coated with the full-length Stx2a.

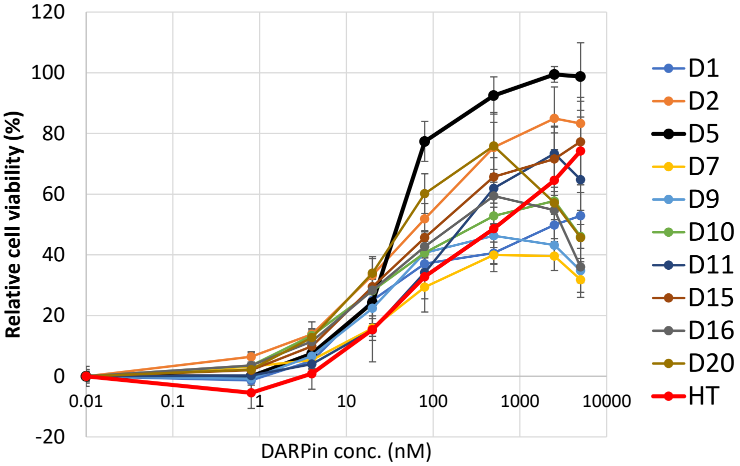


| DARPin Dimer | N-DARPin | C-DARPin | Rank @ 80 nM |
|---|---|---|---|
| D5 | #3 | HT | 1st |
| D20 | #16 | RI* | 2nd |
| D2 | #1 | RI | 3rd |
| D15 | #16 | HT | 4th |
| D16 | #12 | #16 | 5th |
| D10 | #9 | #16 | 6th |
| D9 | #20 | #1 | 7th |
| D1 | #20 | #16 | 8th |
| D11 | 10B | #1 | 9th |
| D7 | 10B | #9 | 10th |

* Variant contains additional point mutations

**Figure S6.** Activity comparison of DARPin dimers.

**Figure S7. Sequence alignment of off-rate mutants.**

| ID | Motif | NCAP / AR1 / AR2 / AR3 / CCAP |
|---|---|---|
| | | 1--------10--------20---------30--------40--------50--------60---------70--------80--------90---------100-------110-------120--------130-------140-------150 |
| | | GKKLLEAARAGQDDEVRILMANGADVNA/XDXXGXTPLHLAAXXGHLEIVEVLLKXGADVNA/XDXXGXTPLHLAAXXGHLEIVEVLLKXGADVNA/XDXXGXTPLHLAAXXGHLEIVEVLLKXGADVNA/QDKFGKTAFDISIDNGNEDLAEILQ |
| | RI | GKKLLEAARAGQDDEVRILMANGADVNA/GDPFGFTPLHLAAMFGHLEIVEVLLKNGADVNA/HDEYGFTPLHLAAVVGHLEIVEVLLKNGADVNA/CDNQGGTPLHLAARIGHLEIVEVLLKHGADVNA/QDKFGKTAFDISIDNGNEDLAEILQ |
| | HT | GKKLLEAARAGQDDEVRILMANGADVNA/GDPFGFTPLHLAAMFGHLEIVEVLLKNGADVNA/HDEYGFTPLHLAAVVGHLEIVEVLLKNGADVNA/CDNQGGTPLHLAAHTGHLEIVEVLLKHGADVNA/QDKFGKTAFDISIDNGNEDLAEILQ |
| #1 | #1-A4 | ----------------------------/---------------------------------/-E-----------------------N-------/-------------HT------------------/--------------I---------- |
| #2 | #1-A10 | -----V----------------------/---------------------------------/-E-------------------------------/-------------HT------------------/------------------------- |
| #3 | #2-A9 | ----------------------------/---------------------------------/-E-------------S-M---------------/-------------HT------------------/------------------------- |
| #4 | #2-G5 | ----------------------------/---------------------------------/-E-------------------------------/-------------RM----M-------------/------------------------- |
| #5 | #2-H7 | ----------------------------/---------------------------------/-E-------------------------------/-------------RI------------------/------------------------- |
| #6 | #2-H8 | -------------------V--------/---------------------------------/-E-----------------------R-------/-------------HT------------------/------------------------- |
| #7 | #3-A6 | -------------------V--------/---------------------------------/-E-----------------------R-------/-------------HT------------------/------------------------- |
| #8 | #3-A7 | ----------------------------/---------------------------------/-E-------------------------------/-------------HT------------------/------------------------- |
| #9 | #3-A8 | ----------------------------/---------------------------------/-E-------------------------------/-------------HT------------------/------------------------- |
| #10 | #3-A10 | ----------------------------/---------------------------------/-E--------------L----------------/-------------HT------------------/-------------GY---------- |
| #11 | #3-B2 | ---------------------------V/---------------------------------/-E-------------------------------/-------------HT------------------/------------------------- |
| #12 | #3-C4 | ----------------------------/-------------LY------------------/-E-------------------------------/-------------HT------------------/------------------------- |
| #13 | #3-D10 | ----------------------------/--------------Y------------------/-E-------------------------------/-------------HT------------------/--------Y---------------- |
| #14 | #3-D12 | --------------V-------------/--------------------A---S--------/-E----------------G--------------/-------------RI------------------/------------------------- |
| #15 | #3-E9 | ----------------------------/---------------------------------/-E-------------------------------/-------------HT------------------/------------------------- |
| #16 | #3-E10 | -----D---T------------------/-------------L-------------------/-E-------------------------------/-------------RI-----------Q------/------------------------- |
| #17 | #3-E11 | ----------------------------/-------------------------M-------/-E-------------------G-----------/S------------RI------------------/------------------------- |
| #18 | #3-F8 | ----------------------------/------------------G--------------/-E-------------------------------/-------------RI------------------/--------------I---------- |
| #19 | #3-F12 | ----------------------------/----------F----------------------/-E-------------------------------/-------------HT------------------/--------------I---------- |
| #20 | #4-A2 | -----------------T----------/--------------Y------------------/-E-------------------------------/-------------HT------------------/------------------------ |
| #21 | #4-A10 | ----------------------------/---------------------------------/-E-------------------------------/-------------HT------------------/------------------------- |
| #22 | #4-B5 | ----------------------------/-----------------------------G---/-E------------------L------------/R-----S------HT-----------R------/------------------------- |
| #23 | #4-E1 | ----------------------------/C--------------------------------/-E-------------------------------/-------------HT------------------/------------------------- |
| #24 | #4-F2 | ----------------------------/---------------------------------/-E-----------------------N-------/-------------HT------------------/------------------------- |
| #25 | #4-F9 | ----------------------------/------------------------M-----A--/-E-------------------------------/-------------HT------------------/------------------------- |
| #26 | #4-G7 | ----------------------------/---------------------------------/-E-------------------------------/------------SHT------------------/--------------T---------- |
| #27 | #4-H5 | -----------------F----------/------------------------------E--/-E-------------------------------/-------------HT------------------/------------------------- |
| #28 | #4-H7 | ----------------------------/--------------Y------------------/-E-------------------------------/-------------RI------------------/------------------------- |
| Syn | SHT | -------------------V--------/-------------LY------------------/-E-------------S-M-------N-------/R-----S-----SHT-----------Q------/--------Y-----I---------- |

**A**

```
Stx2a-A      MKCILFKWVLCLLLGFSSVSYSREFTIDFSTQQSYVSSLNSIRTEISTPLEHISQGTTSV    60
Stx2c-A      MKCILFKWVLCLLLGFSSVSYSREFTIDFSTQQSYVSSLNSIRTEISTPLEHISQGTTSV    60
             ************************************************************

Stx2a-A      SVINHTPPGSYFAVDIRGLDVYQARFDHLRLIIEQNNLYVAGFVNTATNTFYRFSDFTHI    120
Stx2c-A      SVINHTPPGSYFAVDIRGLDVYQARFDHLRLIIEQNNLYVAGFVNTATNTFYRFSDFTHI    120
             ************************************************************

Stx2a-A      SVPGVTTVSMTTDSSYTTLQRVAALERSGMQISRHSLVSSYLALMEFSGNTMTRDASRAV    180
Stx2c-A      SVPGVTTVSMTTDSSYTTLQRVAALERSGMQISRHSLVSSYLALMEFSGNTMTRDASRAV    180
             ************************************************************

Stx2a-A      LRFVTVTAEALRFRQIQREFRQALSETAPVYTMTPGDVDLTLNWGRISNVLPEYRGEDGV    240
Stx2c-A      LRFVTVTAEALRFRQIQREFRQALSETAPVYTMTPGDVDLTLNWGRISNVLPEYRGEDGV    240
             ************************************************************

Stx2a-A      RVGRISFNNISAILGTVAVILNCHHQGARSVRAVNEESQPECQITGDRPVIKINNTLWES    300
Stx2c-A      RVGRISFNNISAILGTVAVILNCHHQGARSVRAVNEDSQPECQITGDRPVIKINNTLWES    300
             ************************************:***********************

Stx2a-A      NTAAAFLNRKSQFLYTTGK   319
Stx2c-A      NTAAAFLNRKSQFLYTTGK   319
             ************ *****:


Stx2a-B      MKKMFMAVLFALASVNAMAADCAKGKIEFSKYNEDDTFTVKVDGKEYWTSRWNLQPLLQS    60
Stx2c-B      MKKMFMAVLFALVSVNAMAADCAKGKIEFSKYNENDTFTVKVAGKEYWTSRWNLQPLLQS    60
             ************.*********************:******* *****************

Stx2a-B      AQLTGMTVTIKSSTCESGSGFAEVQFNND  89
Stx2c-B      AQLTGMTVTIKSSTCESGSGFAEVQFNND  89
             *****************************
```

**B**

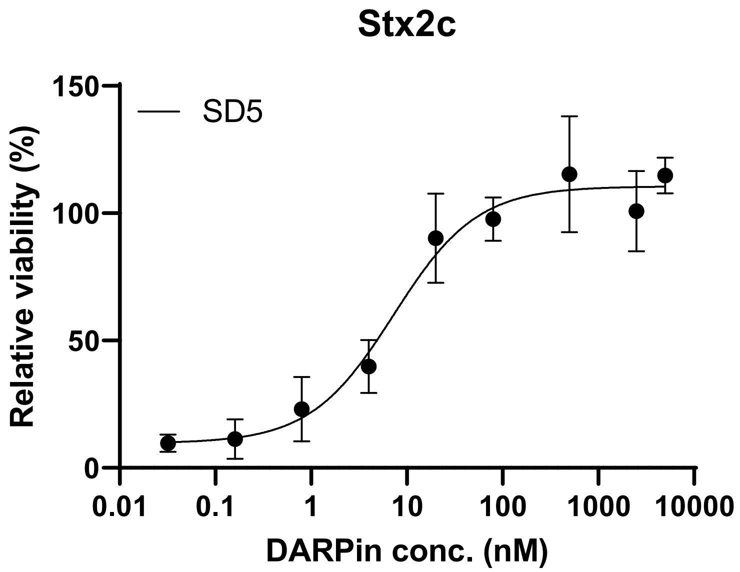


**Figure S8. (A) Sequence alignment of Stx2a and Stx2c. (B) Protection of Vero cells by SD5.** Vero E6 cells ($1.5x10^3$ cells/well) were incubated with 60 pg/mL Stx2c and the appropriately diluted DARPin for 3 days. Error bars represent the standard deviation of two independent experiments performed in duplicate.

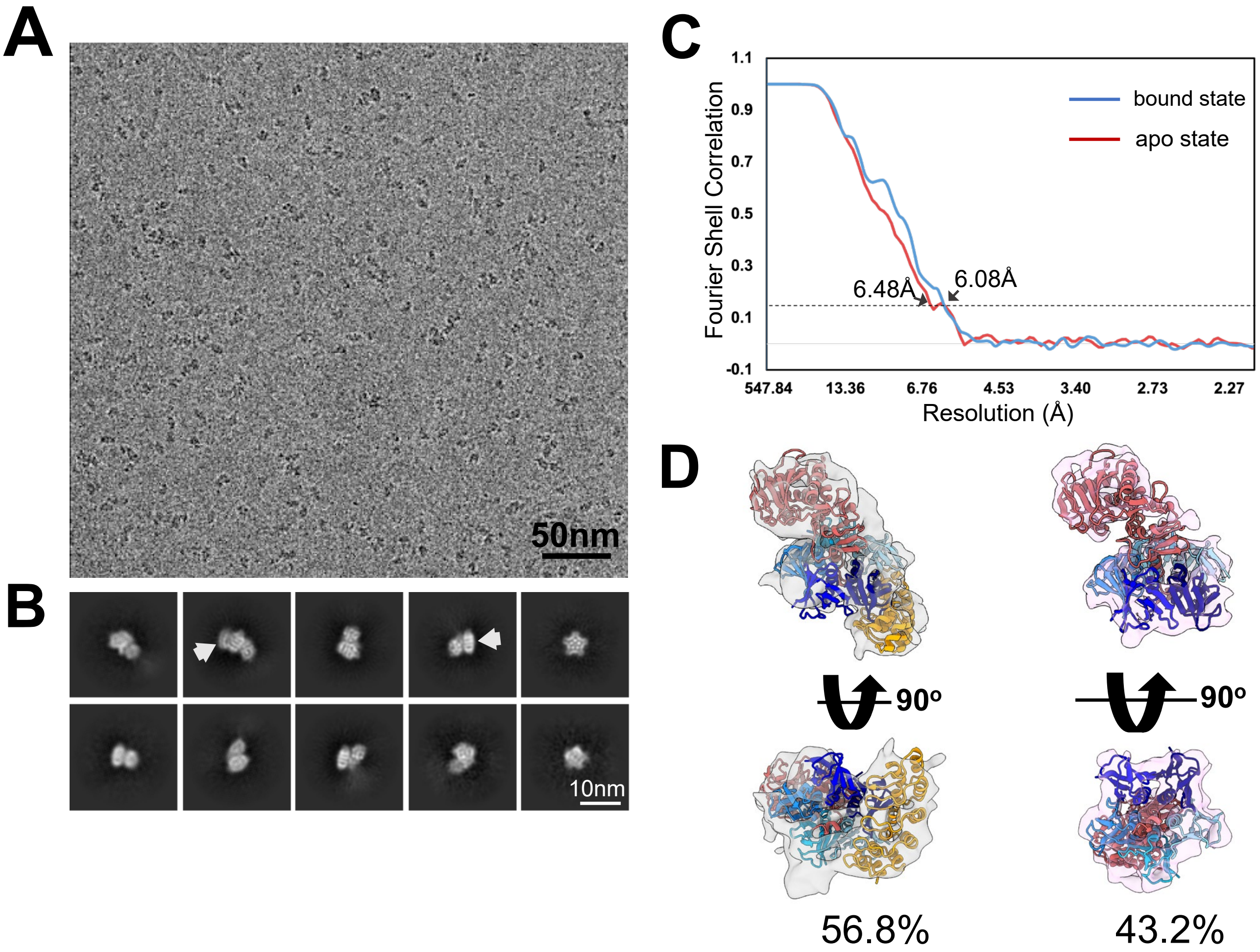


**Figure S9.** Cryo-EM analysis on Stx2a and DARPin #3 complex. **(A)** Representative micrograph of the cryo-EM data. **(B)** Representative 2D classification of the complex. The white arrows highlight the extra density of DARPin. **(C)** FSC of the bound-state map and apo-state map. **(D)** The density maps of the bound state (left) and the apo state (right) with the refined models docked in. The A subunit is colored red. The B subunit is colored blue. The DARPin is colored orange.

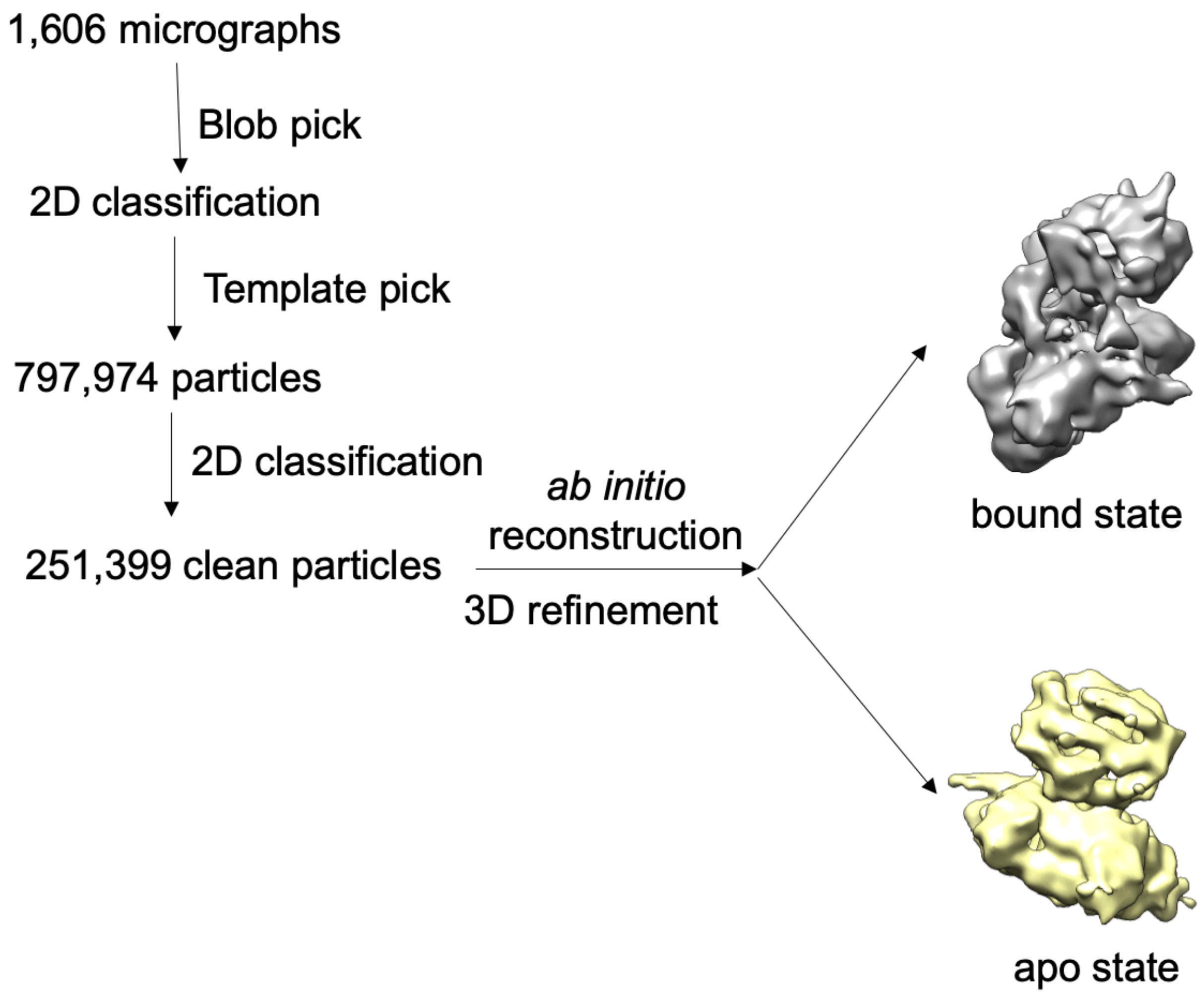


**Figure S10.** Data processing for the complex between Stx2a and DARPin #3.
